# Association between Ambient Air Pollution and Diabetes Mellitus in Europe and North America: Systematic Review and Meta-Analysis

**DOI:** 10.1289/ehp.1307823

**Published:** 2015-01-27

**Authors:** Ikenna C. Eze, Lars G. Hemkens, Heiner C. Bucher, Barbara Hoffmann, Christian Schindler, Nino Künzli, Tamara Schikowski, Nicole M. Probst-Hensch

**Affiliations:** 1 Swiss Tropical and Public Health Institute, Basel, Switzerland; 2 University of Basel, Basel, Switzerland; 3 Basel Institute for Clinical Epidemiology and Biostatistics, University Hospital Basel, Basel, Switzerland; 4 IUF-Leibniz Research Institute for Environmental Medicine, Düsseldorf, Germany; 5 Medical Faculty, Heinrich Heine University of Düsseldorf, Düsseldorf, Germany

## Abstract

**Background:**

Air pollution is hypothesized to be a risk factor for diabetes. Epidemiological evidence is inconsistent and has not been systematically evaluated.

**Objectives:**

We systematically reviewed epidemiological evidence on the association between air pollution and diabetes, and synthesized results of studies on type 2 diabetes mellitus (T2DM).

**Methods:**

We systematically searched electronic literature databases (last search, 29 April 2014) for studies reporting the association between air pollution (particle concentration or traffic exposure) and diabetes (type 1, type 2, or gestational). We systematically evaluated risk of bias and role of potential confounders in all studies. We synthesized reported associations with T2DM in meta-analyses using random-effects models and conducted various sensitivity analyses.

**Results:**

We included 13 studies (8 on T2DM, 2 on type 1, 3 on gestational diabetes), all conducted in Europe or North America. Five studies were longitudinal, 5 cross-sectional, 2 case–control, and 1 ecologic. Risk of bias, air pollution assessment, and confounder control varied across studies. Dose–response effects were not reported. Meta-analyses of 3 studies on PM_2.5_ (particulate matter ≤ 2.5 μm in diameter) and 4 studies on NO_2_ (nitrogen dioxide) showed increased risk of T2DM by 8–10% per 10-μg/m^3^ increase in exposure [PM_2.5_: 1.10 (95% CI: 1.02, 1.18); NO_2_: 1.08 (95% CI: 1.00, 1.17)]. Associations were stronger in females. Sensitivity analyses showed similar results.

**Conclusion:**

Existing evidence indicates a positive association of air pollution and T2DM risk, albeit there is high risk of bias. High-quality studies assessing dose–response effects are needed. Research should be expanded to developing countries where outdoor and indoor air pollution are high.

**Citation:**

Eze IC, Hemkens LG, Bucher HC, Hoffmann B, Schindler C, Künzli N, Schilowski T, Probst-Hensch NM. 2015. Association between ambient air pollution and diabetes mellitus in Europe and North America: systematic review and meta-analysis. Environ Health Perspect 123:381–389; http://dx.doi.org/10.1289/ehp.1307823

## Introduction

Ambient air pollution ranks high among risk factors for the global burden of disease (Lim et al. 2012), and is linked to several chronic noncommunicable conditions such as cardiovascular diseases (Bauer et al. 2010; Brook et al. 2010; Künzli et al. 2010), asthma (Bui et al. 2013; Jacquemin et al. 2012; Künzli et al. 2009), chronic obstructive pulmonary diseases (COPD) (Andersen et al. 2011; Schikowski et al. 2014; Zanobetti et al. 2008), and cancers including lung (Raaschou-Nielsen et al. 2013a), cervical, and brain cancers (Raaschou-Nielsen et al. 2011). Persons with type 2 diabetes mellitus (T2DM) are at increased risk to develop micro- and macrovascular diseases and reduced lung function (Jones et al. 2014; Kinney et al. 2014). Air pollution has also been shown to be more detrimental to diabetic patients, worsening their clinical outcomes (O’Neill et al. 2005; Raaschou-Nielsen et al. 2013b; Whitsel et al. 2009; Zanobetti and Schwartz 2001).

More recent evidence is supportive of an air pollution effect on diabetes risk. Experimental evidence show that possible pathways may include endothelial dysfunction, overactivity of the sympathetic nervous system (Rajagopalan and Brook 2012), immune response alterations in visceral adipose tissues; endoplasmic reticulum stress resulting in alterations in insulin transduction (Sun et al. 2009), insulin sensitivity, and glucose metabolism; and alterations in mitochondria and brown adipocytes (Liu et al. 2013; Rajagopalan and Brook 2012).

Papazafiropoulou et al. (2011) systematically reviewed the etiologic association between environmental pollution and diabetes, taking into account studies on organic pollutants and secondary effects of air pollution on diabetic patients published up to November 2010. They described a positive association between environmental pollution and prevalent diabetes, as well as increased morbidity and mortality among diabetic patients. A number of pertinent studies have been published since this review, and thus far there is, to the best of our knowledge, no meta-analysis of the available evidence. We therefore systematically identified and reviewed the epidemiological evidence on the association between air pollution and diabetes mellitus, and synthesized the results of studies on the association with T2DM.

## Methods

*Search strategy*. We systematically searched electronic literature databases [MEDLINE (http://www.nlm.nih.gov/bsd/pmresources.html), EMBASE (https://www.embase.com), and ISI Web of Science (http://www.webofknowledge.com)] for pertinent literature published up to 3 February 2014. Terms used in this search included “air pollution,” “air pollutants,” “particulate matter,” “PM_10_,” “PM_2.5_,” “nitrogen dioxide,” “NO_2_,” “NO_x_,” “ozone,” “soot,” “smog,” “diabetes mellitus,” “diabetes,” “T1DM,” “T2DM,” “type 1 DM,” “type 2 DM,” “IDDM,” “NIDDM,” alone and in combination. We applied no filters for study designs. Reference lists of eligible articles were searched for further pertinent articles. After de-duplication, titles and abstracts were screened for eligibility and potentially relevant articles were retrieved as full texts. Screening was performed independently by two reviewers and any discrepancies were resolved by discussion.

*Inclusion and exclusion criteria*. We included only original research published in English as a full publication in a peer-reviewed journal. We accepted any type of study design. In eligible studies, the definition of air pollution and diabetes mellitus had to be clearly stated. Air pollution had to be outdoor (ambient, including traffic-related), and we accepted any type of assessment including particle concentration in the air or indicators of long-term traffic exposure. Diabetes mellitus had to be physician diagnosed or based on the use of antidiabetic medications. We included any type of diabetes mellitus (type 1, type 2, and gestational). Eligible studies had to report quantitative measures of association between air pollution and diabetes mellitus, and their 95% confidence intervals (CIs) (or enough data to allow derivation of this association). We excluded studies that were based on the effect of blood markers, and not clearly defining clinical outcomes. Studies testing only whether diabetes status would modify the association between air pollution and health outcomes were not considered in this review. Animal studies were excluded.

For the meta-analysis, only studies on individual type 2 diabetes risk were included. We included all studies that quantified particle concentrations as “per ... μg/m^3^” or “ppb.” If the diabetes type was not clearly stated, we considered diagnoses of diabetes in nonpregnant adults (≥ 18 years age) as diagnoses of T2DM because > 90% of new diagnoses of adult diabetes is type 2 diabetes (Alberti and Zimmet 1998).

*Data extraction*. We extracted the following data from the eligible studies: year of study, study setting, study design, year of publication, population demographics, study definition of diabetes and assessment of air pollution exposure, confounder adjustments, and effect modification assessments. We extracted data on the effect estimates (unadjusted and final model) of the association (and their 95% CIs) between air pollution and diabetes.

Data were extracted independently by two reviewers and disagreements were resolved by discussion.

*Meta-analysis*. We used random-effects models to synthesize the associations between air pollution and T2DM (Lau et al. 1997). Random-effect models give more weight to smaller studies and have typically wider CIs because in addition to the within-study variance, they also consider potential variation between the true effects that all included studies estimate. We used fixed-effects models (which assume that all studies share a common true effect) in a sensitivity analysis.

We used risk ratios as measure of association across all studies. When hazard ratios and incidence risk ratios were reported, we directly considered them as risk ratios. Because diabetes is not very common, we considered reported odds ratios as equivalent to risk ratios. For studies with estimates of association from multiple particle concentration sources, we chose the estimates modelled at participants’ residences (land-use regression, kriging, or satellite-based estimates). We used the effect estimates reported by the study authors as “main model” or “fully adjusted model.” We used estimates of association and their standard errors reported as “per 10 μg/m^3^” of exposure and we converted other reported quantities or units where necessary.

We described the between-study heterogeneity using the *I*^2^ metric and the between-studies’ variance using Tau^2^. We assessed publication bias using the Egger’s test for asymmetry (Egger et al. 1997). We conducted sensitivity analyses including only studies that *a*) measured air pollution exposure before DM diagnosis, *b*) comprised both males and females, and *c*) were longitudinal, and we applied a fixed-effects analysis. All analyses were performed with Stata version 12 (StataCorp, College Station, TX, USA) using the “metan” command. *p-*Values were two-tailed and *p* < 0.05 was considered nominally statistically significant.

For reporting, we followed the Meta-analysis Of Observational Studies in Epidemiology (Stroup et al. 2000) and the Preferred Reporting Items for Systematic Reviews and Meta-Analysis (Moher et al. 2010) guidelines.

## Results

**Figure 1:**
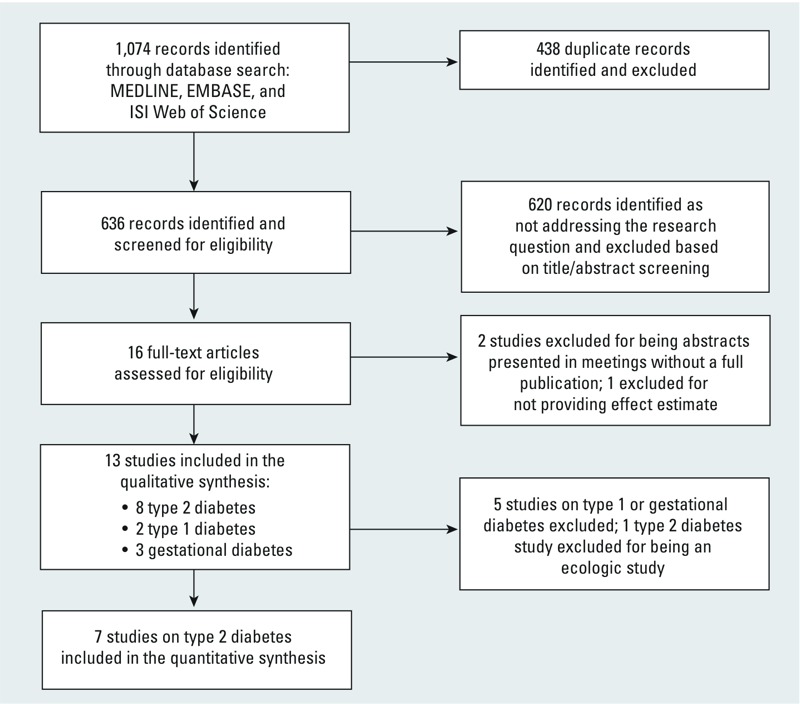
Results of systematic literature search.

The database search yielded 636 records after de-duplication, which were screened on title/abstract level for eligibility (Figure 1). Sixteen potentially eligible articles were screened on full-text level, and 3 were excluded (Figure 1). Thirteen studies were included (Table 1). There were 5 longitudinal studies (Andersen et al. 2012; Chen et al. 2013; Coogan et al. 2012; Krämer et al. 2010; Puett et al. 2011), 5 cross-sectional studies (Brook et al. 2008; Dijkema et al. 2011; Fleisch et al. 2014; Malmqvist et al. 2013; van den Hooven et al. 2009), 2 case–control studies (Hathout et al. 2002, 2006), and 1 ecologic study (Pearson et al. 2010). Two studies were on type 1 diabetes (Hathout et al. 2002, 2006); 3 studies on gestational diabetes (GDM) (Fleisch et al. 2014; Malmqvist et al. 2013; van den Hooven et al. 2009), and 8 studies on T2DM (Andersen et al. 2012; Brook et al. 2008; Chen et al. 2013; Coogan et al. 2012; Dijkema et al. 2011; Krämer et al. 2010; Pearson et al. 2010; Puett et al. 2011). Seven non-ecological studies on T2DM were selected for quantitative synthesis (with the exclusion of Pearson et al. 2010). Air pollution estimates from these studies were based on land-use regression (Andersen et al. 2012; Brook et al. 2008; Dijkema et al. 2011; Krämer et al. 2010; Puett et al. 2011), kriging (Coogan et al. 2012), and satellite-derived estimates (Chen et al. 2013). All studies were conducted in Europe or North America. Tables 1 and 2 and Supplemental Material, Table S1, provide an overview of the 13 eligible studies. Table 3 summarizes the data reported in the studies synthesized in meta-analyses.

**Table 1:** Characteristics of the studies on the relationship between air pollution and diabetes mellitus.

Source	Location	Years of study	Study design and duration of follow-up	Population (*n*) and age (years) of participants
Krämer et al. 2010^*a*^	Ruhrgebiet, Germany	1990–2006	Longitudinal: Study on the Influence of Air Pollution on Lung Inflammation and AgingFollow-up: 16 years	*n* = 1,775 Caucasian women without T2DM at baseline, 54–55 years
Andersen et al. 2012^*a*^	Copenhagen and Aarhus, Denmark	(1993–1997) –2006	Longitudinal: Danish Diet, Cancer and Health cohortFollow-up: 9.7 years	*n* = 51,818 Caucasians without DM at baseline, 50–65 years
Puett et al. 2011^*a*^	Metropolitan Statistical Areas (MSA) in north-eastern and midwestern states of USA	1989–2009	Longitudinal, with 2 cohorts: Nurses’ Health Study and Health Professionals Follow-up StudyFollow-up: 20 years	*n* = 74,412 female nurses 30–55 years and 15,048 male health professionals 40–75 years, without T2DM at baseline
Coogan et al. 2012^*a*^	Los Angeles, California, USA	1995–2005	Longitudinal: Black Women’s Health StudyFollow-up: 10 years	*n* = 3,992 African-American women, without DM at baseline and 21–69 years
Chen et al. 2013^*a*^	Ontario, Canada	(1996–2005) –2010	LongitudinalFollow-up: 8 years	*n* = 62,012 Canadians without DM, ≥ 35 years
Brook et al. 2008^*a*^	Hamilton and Toronto, Ontario, Canada	1992–1999	Cross-sectional	*n* = 7,634 patients who attended two respiratory clinics in Hamilton and Toronto, ≥ 40 years
van den Hooven et al. 2009	Rotterdam, Netherlands	2002–2006	Cross-sectional: Generation R study	*n* = 7,399 pregnant women, who had delivery date in the study period, 21–38 years
Dijkema et al. 2011	Westfriesland, Netherlands	1998–2000	Cross-sectional: Hoorn Screening Study for T2DM	*n* = 8,018 Caucasian residents, 50–75 years
Malmqvist et al. 2013	Scania, Sweden	1999–2005	Cross-sectional: The Swedish Medical Birth Registry.	*n* = 81,110 women who had singleton deliveries during the study period
Hathout et al. 2006	California, USA	2002–2003	Case–controlFollow-up: retrospectively from birth until diagnosis of T1DM	*n* = 402 children (102 with T1DM and 300 age-matched controls), 1–12 years, receiving care at Loma Linda University Pediatric Center
Hathout et al. 2002	California, USA	2002	Case–controlFollow-up: retrospectively from birth until diagnosis of T1DM	*n* = 100 children (61 cases: 30 had onset ≤ 5 years and 31 > 5 years) (39 age-matched controls: 19 were ≤ 5 years and 20 were > 5 years) receiving care at Loma Linda University Pediatric Center
Fleisch et al. 2014	Boston, Massachusetts, USA	1999–2002	Cross-sectional: Project Viva Cohort	*n* = 2,093 second-trimester pregnant women without known diabetes
Pearson et al. 2010	USA	2004–2005	Ecologic	*n* = 3,082 counties of USA
Abbreviations: T2DM, type 1 diabetes mellitus; T2DM, type 2 diabetes mellitus. ^***a***^Included in meta-analysis.				

**Table 2:** Exposure and outcome definitions.

Source	Outcome	Definition of outcome	Exposure	Definition of exposure	Exposure estimates
Krämer et al. 2010^*a*^	Incident T2DM	Self-reported, physician-diagnosed T2DM	PM_10_, PM, PM_2.5_, NO_2_, and traffic exposure	5-year means of PM_10_ and NO_2_ in an 8-km grid from monitoring stations, before baseline	Median (25th–75th percentile) Monitoring stations (μg/m^3^): PM_10_: 46.9 (44–54.1) NO_2_: 41.7 (23.3–48.2)
				Traffic PM and NO_2_ in a 1-km grid, in 1 year, from emission inventory	Traffic emission inventory (tons/year/km^2^): PM: 0.54 (0.22–1.09)
				Traffic PM_2.5_ and NO_2_^*b*^ from a (1-year measurement) LUR model. Distance from the next major road with > 10,000 cars per day	NO_2_: 12 (5.4–24.4) LUR soot (10^–5 ^m): 1.89 (1.67–2.06)NO_2_ (μg/m^3^): 34.5 (23.8–38.8) % participants living < 100 m from busy road: 15.8
Andersen et al. 2012^*a*^	Incident DM	Confirmed DM cases from the Danish National Diabetes Register	NO_2_, NO_x_, traffic exposure	35^*b*^- and 15-year mean levels of NO_2_ and NO_x_, from the Danish AirGIS model before baseline	Median (IQR) 35-year NO_2_ and NO_x_ (μg/m^3^): 14.5 (4.9) and 20.9 (11.4) 15-year NO_2_ and NO_x_ (μg/m^3^): 15.3 (5.6) and 22.1 (12)
				1-year mean NO_2_ and NO_x_ at baseline	1-year NO_2_ and NO_x_ at baseline (μg/m^3^): 15.4 (5.6) and 20.3 (10.9)
				1-year mean NO_2_ and NO_x_ at follow-up	1-year NO_2_ and NO_x_ at follow-up (μg/m^3^): 15.2 (5.7) and 21.5 (12)
				Major road (with annual traffic density of ≥ 10,000) within 50 m of residence.	% major road within 50 m: 8.1
				Traffic load within 100 m of residence (10^3^ vehicles/km/day)	Traffic load within 100 m (10^3^ vehicles/km/day): 0.34 (1.3)
Puett et al. 2011^*a*^	Incident T2DM	DM according to the National Diabetes Data Group Criteria^*c*^	PM_2.5_, PM_10_, PM_10–2.5_	Average PM_2.5_^*b*^, PM_10_, and PM_10–2.5_ concentrations, from LUR model, 12 months before diagnosis	Mean ± SD PM_2.5_ (μg/m^3^): 18.3 ± 3.1 for HPFS and 17.5 ± 2.7 for NHSPM_10_ (μg/m^3^): 28.5 ± 5.5 for HPFS and 26.9 ± 4.8 for NHS PM_10–2.5_ (μg/m^3^): 10.3 ± 3.3 for HPFS and 9.4 ± 2.9 for NHS
Coogan et al. 2012^*a*^	Incident T2DM	Self-reported, physician-diagnosed T2DM	PM_2.5_, NO_x_, traffic exposure	1-year mean PM_2.5_^*b*^ during follow-up, assigned by kriging model	Mean ± SDPM_2.5_ (μg/m^3^): 20.7 ± 2.1 Median (25th–75th percentile) PM_2.5_ (μg/m^3^): 21.1 (20.3–21.6)
				1-year mean NO_x_ the year after follow-up, assigned by LUR model	Mean ± SD NO_x_ (ppb): 43.3 ± 11 Median (25th–75th percentile) NO_x_ (ppb): 41.6 (36.9–49.2)
Chen et al. 2013^*a*^	Incident DM	Physician-diagnosed DM from Ontario database	PM_2.5_	6-year mean PM_2.5_^*b*^ during baseline/ follow-up, obtained from satellite-based estimates at 10 x 10 km resolution	Mean (range) PM_2.5_ (μg/m^3^): 10.6 (2.6–19.1)
Brook et al. 2008^*a*^	Prevalent DM	Physician-diagnosed DM from Ontario Health Insurance Plan and Ontario Health Discharge Database	NO_2_	NO_2_^*b*^ assigned by LUR models developed from mean field measurements within 3 years, from Hamilton and Toronto, Ontario, Canada	Median (25th–75th percentile) NO_2_ (ppb) Males: Hamilton: 15.2 (13.9–17.1); Toronto: 23 (20.8–25) Females:Hamilton: 15.3 (14–17); Toronto: 22.9 (20.8–24.7)
van den Hooven et al. 2009	Prevalent gestational DM (GDM)	GDM diagnosed according to the Dutch midwifery and obstetric guidelines	Traffic exposure	Distance-weighted traffic density (DWTD) within a 150-m radius around residence (vehicles/24 hr × m)	Median (P25–P75) DWTD (vehicles/24 hr × m): 5.5 × 10^5^ (1.6 × 10^5 ^– 1.2 × 10^6^)
				Proximity to a major road (> 10,000 vehicles/day)	Proximity to a major road (m): 143 (74–225)
Dijkema et al. 2011	Prevalent T2DM	Self-reported physician-diagnosed T2DM. Laboratory-based diagnosis for undetected cases	NO_2_, traffic exposure	1-year mean NO_2_ assigned by LUR model	Median (25th–75th percentile) NO_2_ (μg/m^3^): 15.2 (14.2–16.5)
				Distance to the nearest main road (≥ 5,000 vehicles/day)	Distance to nearest main road (m): 140 (74–220)
				Traffic flow at the nearest main road (vehicles/24 hr)	Traffic flow at the nearest main road (10^3^ vehicles/24 hr): 7.31 (5.87–9.67)
				Total traffic per 24 hr on all roads within a 250-m circular buffer around the address	Traffic within 250-m buffer (10^3^ vehicles/24 hr): 680 (516–882)
Malmqvist et al. 2013	Prevalent GDM	GDM as defined in the Swedish Medical Birth Registry	NO_x_, traffic exposure	Monthly and trimester means of NO_x_ assigned by dispersion modeling at a spatial resolution of 500 × 500 m over the duration of the pregnancy	Quartiles of NO_x_ exposure (μg/m^3^): Q1: 2.5–8.9 Q2: 9.0–14.1 Q3: 14.2–22.6 Q4: > 22.7
				Traffic density within a 200-m radius	Categories of traffic density within 200 m (vehicles/min):1: no road 2: < 2 3: 2–5 4: 5–10 5: > 10
Hathout et al. 2006	Prevalent T1DM	Physician-diagnosed T1DM from the database of Loma Linda University Pediatric Center	O_3_, NO_2_, SO_2_, SO_4_, and PM_10_	Average monthly pollutant exposure (obtained from the U.S. EPA and California Air Resources Board) from birth until diagnosis for cases and until enrollment for controls, assigned to residential ZIP codes	Mean (95% CI) For cases: O_3_: 29.4 (28, 30.8) ppb SO_4_: 3.6 (3.4, 3.87) μg/m^3 ^SO_2_: 1.6 (1.41, 1.75) ppb NO_2_: 30.3 (28.4, 32.3) ppb PM_10_: 48.6 (45.9, 51.3) μg/m^3^For controls: O_3_: 25.8 (25.2, 26.3) ppb SO_4_: 3.3 (3.2, 3.36) μg/m^3^ SO_2_: 1.5 (1.42, 1.5) ppb NO_2_: 29.7 (29.1, 30.4) ppb PM_10_: 47.4 (46.3, 48.5) μg/m^3^
Hathout et al. 2002	Prevalent T1DM	Physician-diagnosed T1DM from the database of Loma Linda University Pediatric Center	O_3_, NO_2_, SO_2_, SO_4_, and PM_10_	Average monthly pollutant exposure (obtained from the U.S. EPA and California Air Resources Board) from birth until diagnosis for cases and until enrollment for controls, assigned to residential ZIP codes	Mean ± SDFor cases: O_3_: 32.5 ± 5.22 ppb SO_4_: 5.52 ± 0.75 μg/m^3 ^SO_2_: 0.67 ± 0.55 pphm NO_2_: 23.7 ± 7.91 ppb PM_10_: 59.3 ± 12.9 μg/m^3^ For controls: O_3_: 26.7 ± 9.6 ppb SO_4_: 5.88 ± 1.04 μg/m^3 ^SO_2_: 1.29 ± 0.92 pphm NO_2_: 24.7 ± 7.26 ppb PM_10_: 49.6 ± 14.7 μg/m^3^
Fleisch et al. 2014	Prevalent GDM	Failed GCT^*d*^ with ≥ 2 high values on the OGTT^*e*^		PM_2.5_ and black carbon from central sites within 40 km of residence	Mean ± SD From central sites: PM_2.5_: 10.9 ± 1.4 μg/m^3^ Black carbon: 0.9 ± 0.1 μg/m^3^
				PM_2.5_ and black carbon from spatio-temporal models	From spatiotemporal models: PM_2.5_: 11.9 ± 1.4 μg/m^3 ^Black carbon: 0.7 ± 0.2 μg/m^3^
				Neighborhood traffic density [(vehicles/day) × km] within 100 m	Traffic density: 1,621 ± 2,234 (vehicles/day × km)
				Home roadway proximity (≤ 200 m)	Roadway proximity: 281 ± 13
Pearson et al. 2010	Prevalent DM	County-level DM prevalence from the Centers for Disease Control and Prevention	PM_2.5_	County annual mean level of PM_2.5_ obtained from the U.S. EPA as 36-km model, 12-km model, and surface monitor data	PM_2.5_ (μg/m^3^):2004: 36-km model: Q1 mean = 7.71; Q4 mean = 12.11 12-km model: Q1 mean = 7.78; Q4 mean = 11.77 Ground data: Q1 mean = 9.43; Q4 mean = 12.69 2005: 36-km model: Q1 mean = 7.69; Q4 mean = 12.75 12-km model: Q1 mean = 8.41; Q4 mean = 12.38 Ground data: Q1 mean = 9.51; Q4 mean = 13.65
Abbreviations: AirGIS, Air geographic information system; DM, diabetes mellitus; DWTD, distance-weighted traffic density; EPA, Environmental Protection Agency; GDM, gestational diabetes mellitus; HPFS, Health Professionals Follow-up Study; LUR, land-use regression; NHS, Nurses’ Health Study; NO_2_, nitrogen dioxide; NO_x_, nitrogen oxides; O_3_, ozone; OGTT, oral glucose tolerance test; PM, particulate matter; PM_10,_ particulate matter ≤ 10 μm in diameter; PM_10–2.5_, particulate matter between 2.5 and 10 μm in diameter; PM_2.5_, particulate matter ≤ 2.5 μm in diameter; SO_2_, sulfur dioxide; SO_4_, sulfate; T1DM, type 1 diabetes mellitus; T2DM, type 2 diabetes mellitus. ^***a***^Studies included in meta-analysis. ^***b***^Air pollution estimates pooled in the meta-analysis. ^***c***^Elevated plasma glucose concentration on at least two different occasions, one or more DM symptoms and a single elevated plasma glucose concentration, or treatment with hypoglycemic medication. ^***d***^Glucose challenge test: serum glucose 1 hr after a non-fasting 50-g oral glucose load. ^***e***^Oral glucose tolerance test: serum glucose 3 hr after a fasting 100-g glucose load.					

**Table 3:** Data synthesized for meta-analysis.

Source	Population	Pollutant	Assignment of individual exposure	Reported fully adjusted estimate (95% CI)^*a*^
Krämer et al. 2010	Females	NO_2_	LUR model	1.42 (1.16, 1.73) per 15 μg/m^3^ of exposure
Andersen et al. 2012	Females	NO_2_	LUR model	1.07 (1.01, 1.13) per 4.9 μg/m^3^ of exposure
	Males	NO_2_	LUR model	1.01 (0.97, 1.07) per 4.9 μg/m^3^ of exposure
	Both	NO_2_	LUR model	1.04 (1.00, 1.08) per 4.9 μg/m^3^ of exposure
Brook et al. 2008	Females	NO_2_	LUR model	1.04 (1.00, 1.08) per 1 ppb of exposure
	Males	NO_2_	LUR model	0.99 (0.95, 1.03) per 1 ppb of exposure
	Both	NO_2_	LUR model	1.015 (0.98, 1.049) per 1 ppb of exposure
Puett et al. 2011	Females	PM_2.5_	LUR model	1.02 (0.94, 1.09) per 4 μg/m^3^ of exposure
	Males	PM_2.5_	LUR model	1.07 (0.92, 1.24) per 4 μg/m^3^ of exposure
	Both	PM_2.5_	LUR model	1.03 (0.96, 1.10) per 4 μg/m^3^ of exposure
Chen et al. 2013	Females	PM_2.5_	Satellite-based estimates	1.17 (1.03, 1.32) per 10 μg/m^3^ of exposure
	Males	PM_2.5_	Satellite-based estimates	1.03 (0.91, 1.16) per 10 μg/m^3^ of exposure
	Both	PM_2.5_	Satellite-based estimates	1.11 (1.02, 1.21) per 10 μg/m^3^ of exposure
Coogan et al. 2012	Females	PM_2.5_	Kriging model	1.63 (0.78, 3.44) per 10 μg/m^3^ of exposure
Dijkema et al. 2011	Females	NO_2_	LUR model	1.03 (0.90, 1.16) per 10 μg/m^3^ of exposure
	Males	NO_2_	LUR model	0.97 (0.87, 1.09) per 10 μg/m^3^ of exposure
	Both	NO_2_	LUR model	1.00 (0.94, 1.06) per 10 μg/m^3^ of exposure
Abbreviations: LUR, land-use regression; NO_2_, nitrogen dioxide; PM_2.5_, particulate matter ≤ 2.5 μm in diameter. ^***a***^All odds ratio, hazard ratio, and incident risk ratio estimates were converted to per 10 μg/m^3^ of exposure for meta-analysis. Estimates from Dijkema et al. (2011) were derived from reported nonlinear estimates.				

In the Supplemental Material, Table S2 provides an overview of potential sources of bias and how they were assessed by the 13 studies. These are discussed in detail below.

*Bias due to outcome assessment*. As shown in Table 2, some studies relied on self-reported, physician-diagnosed DM (Coogan et al. 2012; Dijkema et al. 2011; Krämer et al. 2010), whereas others linked participants to established databases to identify cases (Andersen et al. 2012; Brook et al. 2008; Chen et al. 2013; Hathout et al. 2002, 2006; Malmqvist et al. 2013). Additional steps were taken by some studies with self-reported outcomes to test the validity of the DM diagnosis. These steps included sending a follow-up questionnaire with the same questions about diabetes (Krämer et al. 2010) and confirmation from medical records provided by physicians (Coogan et al. 2012). Dijkema et al. (2011) further tested participants who did not report physician-diagnosed diabetes, to identify undiagnosed cases.

*Bias due to exposure assessment*. The reviewed studies used different approaches to assess exposure of participants to air pollution, including modeled concentrations of various particulate matters, nitrogen oxides (NO_x_), sulfates, ozone, and various proxies to estimate traffic-related pollution, with varying buffer levels. The studies are also heterogeneous with regard to the lag time considered for exposure assessment. Only the Danish cohort (Andersen et al. 2012) assessed the impact of different lag times, albeit with little evidence for substantial differences in effects (see Supplemental Material, Table S1). In the absence of a biological basis for the latency between exposure and diagnosis of diabetes, different lag times should be tested. Overall, the diversity of exposure measurement makes it difficult to compare the reported effect estimates across these studies.

*Bias due to confounder adjustment*. Indoor air pollution and smoking. Beyond adjustment for basic DM risk factors at baseline (see Supplemental Material, Table S2), Krämer et al. (2010) also adjusted for environmental tobacco smoke (ETS), indoor heating with fossil fuels, as well as occupational exposure to dust, fumes and extreme temperatures; Andersen et al. (2012) also adjusted for ETS. One study done in children considered ETS exposure (Hathout et al. 2006).

Demographics, physical activity, and dietary factors. The longitudinal studies uniformly adjusted for age, body mass index (BMI), and sex (when study population includes both sexes). The studies on women did not adjust for dietary factors, and all longitudinal studies but one adjusted for alcohol consumption and physical activity (see Supplemental Material, Table S1). The other studies assessed confounding by age and BMI except the case–control studies, which did not consider the children’s BMI in their models. The GDM studies mostly considered maternal alcohol consumption (but not dietary factors) whereas the cross-sectional T2DM studies did not consider either factor (see Supplemental Material, Table S1).

Socioeconomic status. There was a uniform adjustment for socioeconomic status in all studies, although on different scales. At the individual level, educational attainment as a socioeconomic determinant was most commonly used across studies, and a few studies additionally considered household income and ethnicity (see Supplemental Material, Table S1). Few studies considered spatial socioeconomic confounding in forms of unemployment rate, urban/rural residence, neighborhood income and neighborhood socioeconomic status score (see Supplemental Material, Table S1). Overall, there was sufficient consideration for individual-level socioeconomic status, but the insufficient control of area-level socioeconomic status may increase the risk of bias.

Co-morbidities. Some co-morbidities associated with diabetes may also be associated with air pollution. These co-morbidities may include hypertension, myocardial infarction, stroke, asthma, and chronic obstructive pulmonary disease (Brook et al. 2010; Pelle et al. 2012; Vojtková et al. 2012). The longitudinal studies considered some of these co-morbidities (see Supplemental Material, Table S1). Participants with co-morbidities were not excluded from any T2DM study.

*Effect modification*. Several studies reported stronger effects in women compared with men (Andersen et al. 2012; Brook et al. 2008; Chen et al. 2013; Dijkema et al. 2011). Other subgroups reported with potentially increased susceptibility include subjects with low education (Andersen et al. 2012; Chen et al. 2013; Krämer et al. 2010), COPD (Andersen et al. 2012; Chen et al. 2013), asthma (Andersen et al. 2012), higher waist-to-hip ratio (Andersen et al. 2012), and higher level of subclinical inflammation (Krämer et al. 2010), nonsmokers (Andersen et al. 2012), and subjects < 50 years or > 65 years of age (Chen et al. 2013) (see Supplemental Material, Table S1). No study assessed interaction between different air pollutants, air pollutants and noise, or interaction between air pollutants and genetic polymorphisms.

*Loss to follow-up*. Losses to follow-up and healthy survivor bias present common problems in epidemiological studies. Puett et al. (2011) reported a loss of < 10% in both studied cohorts over 20 years of follow-up, and Coogan et al. (2012) reported < 20% loss of cohort over 10 years of follow-up. The other longitudinal studies did not report losses to follow-up. None of the studies included sensitivity analyses to estimate the effect of the healthy survivor bias.

*Publication bias*. Although selective reporting and publication bias cannot be ruled out, considering a high probability that negative findings will not be published, we found no indication for such sources of bias (*p*-value of Egger’s test > 0.2). Some studies reported negative findings. However, most studies had several markers of air pollution available, and it remains unclear if some markers have been measured but not reported, so some selective reporting may have occurred.

*Meta-analysis of studies reporting the association of air pollution and risk of T2DM.* Results of seven studies reporting on risk of T2DM [three on particulate matter with diameter ≤ 2.5 μm (PM_2.5_) and four on nitrogen dioxide (NO_2_)] were considered for quantitative synthesis. All studies synthesized for PM_2.5_ were longitudinal. For NO_2_, two were longitudinal and two were cross-sectional.

**Figure 2:**
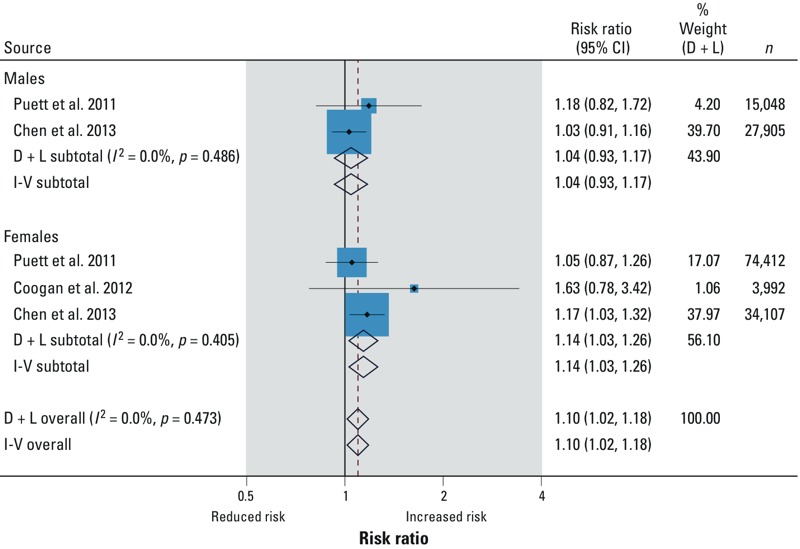
PM_2.5_ and risk of T2DM. Where *I*^2^ is the variation in effect estimates attributable to heterogeneity, D + L (DerSimonian and Laird) overall is the pooled random effect estimate of all studies. I-V (inverse variance) overall is the pooled fixed effects estimate of all studies. Weights are from random-effects analysis. %Weight (D + L) is the weight assigned to each study, based on the inverse of the within- and between-study variance. The size of the blue boxes around the point estimates reflects the weight assigned to each study. The summarized studies were adjusted for age, sex, BMI, smoking, alcohol consumption, and socioeconomic status.

**Figure 3:**
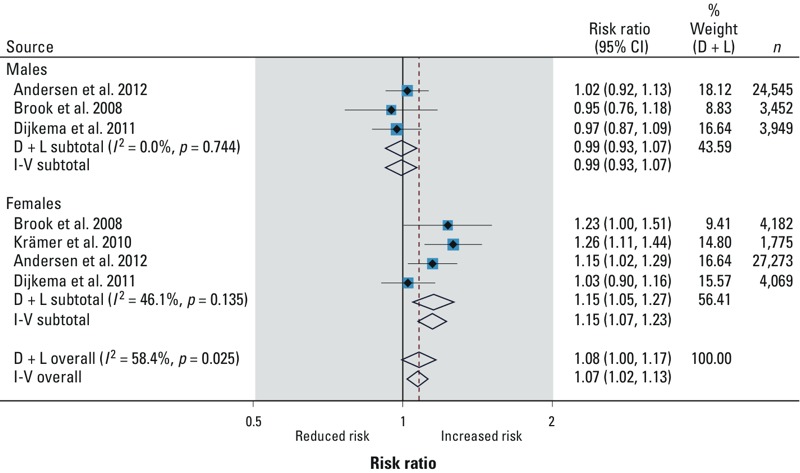
NO_2_ and risk of T2DM. Where *I*^2^ is the variation in effect estimates attributable to heterogeneity, D + L (DerSimonian and Laird) overall is the pooled random-effects estimate of all studies. I-V (inverse variance) overall is the pooled fixed-effects estimate of all studies. Weights are from random-effects analysis. %Weight (D + L) is the weight assigned to each study, based on the inverse of the within- and between-study variance. The size of the blue boxes around the point estimates reflects the weight assigned to each study. The summarized studies were adjusted for age, sex, BMI, smoking, and socioeconomic status.

The pooled relative risks of T2DM per 10-μg/m^3^ increase in exposure to PM_2.5_ (Figure 2) and NO_2_ (Figure 3) were 1.10 (95% CI: 1.02, 1.18) and 1.08 (95% CI: 1.00, 1.17), respectively. The effect was more pronounced in females than in males [NO_2_: 1.15 (95% CI: 1.05, 1.27) vs. 0.99 (95% CI: 0.93, 1.07); PM_2.5_: 1.14 (95% CI: 1.03, 1.26) vs. 1.04 (95% CI: 0.93, 1.17), respectively] per 10-μg/m^3^ increase in exposure. The relative risks were similar across all sensitivity analyses (Table 4). We observed substantial statistical heterogeneity with NO_2_ studies (Table 4). Egger’s test was consistently > 0.2 (*p*-value) in all cases.

**Table 4:** Sensitivity analyses and heterogeneity measures.

Analyses	Population	NO_2_ OR (95% CI)	Heterogeneity measures [*I*^2^ (%); *p*-value; Tau^2^]	PM_2.5_ OR (95% CI)	Heterogeneity measures [*I*^2^ (%); *p*-value; Tau^2^]
Main model (random effects)	Males	0.99 (0.93, 1.07)	0; 0.744; 0	1.04 (0.93, 1.17)	0; 0.486; 0
	Females	1.15 (1.05, 1.27)	46.1; 0.135; 0.0042	1.14 (1.03, 1.26)	0; 0.405; 0
	Overall	1.08 (1.00, 1.17)	58.4; 0.025; 0.0063	1.10 (1.02, 1.18)	0; 0.473; 0
Studies assessing air pollution before DM diagnosis	Males	1.02 (0.92, 1.13)	NA; NA; 0	1.04 (0.93, 1.17)	0; 0.486; 0
	Females	1.20 (1.10, 1.30)	12.5; 0.285; 0.0006	1.13 (1.02, 1.25)	0; 0.344; 0
	Overall	1.12 (1.05, 1.19)	69.8; 0.036; 0.008	1.09 (1.01, 1.18)	0; 0.489; 0
Studies including both men and women	Males	0.99 (0.93, 1.07)	0; 0.744; 0	1.04 (0.93, 1.17)	0; 0.486; 0
	Females	1.11 (1.01, 1.23)	30.2; 0.238; 0.0023	1.13 (1.02, 1.25)	0; 0.344; 0
	Overall	1.05 (0.98, 1.12)	34.9; 0.175; 0.0024	1.09 (1.01, 1.18)	0; 0.489; 0
Only longitudinal studies	Males	1.02 (0.92, 1.13)	NA; NA; 0	1.04 (0.93, 1.17)	0; 0.486; 0
	Females	1.20 (1.10, 1.30)	12.5; 0.285; 0.0006	1.14 (1.03, 1.26)	0; 0.405; 0
	Overall	1.12 (1.05, 1.19)	69.8; 0.036; 0.008	1.10 (1.02, 1.18)	0; 0.473; 0
Meta-analysis using fixed-effects model	Males	1.00 (0.93, 1.07)	0; 0.744	1.04 (0.93, 1.17)	0; 0.486
	Females	1.15 (1.07, 1.23)	46.1; 0.135	1.14 (1.03, 1.26)	0; 0.405
	Overall	1.07 (1.02, 1.13)	58.4; 0.025	1.10 (1.02, 1.18)	0; 0.473
NA, not applicable. *I*^2^ is the proportion of total variability explained by heterogeneity. Tau^2^ is a measure of among-study variance.					

## Discussion

In this systematic review, we considered 13 studies on different types of diabetes. The identified epidemiological evidence is highly diverse: Levels, timing, and assessment of exposure varied, as did the outcome definitions, measures of association, and degree of confounder control. The studies included persons with different age ranges and settings, and some populations included only women. Although there is a risk of bias, the results of the meta-analyses indicate a positive association between traffic-related air pollution and T2DM.

*Pathophysiologic mechanisms of DM–air pollution association*. There is strong evidence supporting the role of inflammation in T2DM (Donath and Shoelson 2011; Sjöholm and Nyström 2006). Chronic activation of inflammatory mechanisms can contribute to chronic insulin resistance and subsequent T2DM. Air pollution has been shown to be inflammatory (Liu et al. 2013; Rajagopalan and Brook 2012). Its potential mechanisms in mediating T2DM include pulmonary and systemic inflammation, directly releasing cytokines, alterations in glucose homeostasis through defective insulin signaling in tissues, immune cells activation in visceral adipose tissues potentiating inflammation (Sun et al. 2009; Xu et al. 2010; Yan et al. 2011), and endoplasmic reticulum stress in the lung and liver in relation with hepatocyte and alveolar cells (Liu et al. 2013; Rajagopalan and Brook 2012). PM_2.5_ also acts as a hypothalamic stressor, inducing peripheral inflammation and abnormalities in glucose metabolism (Liu et al. 2013; Purkayastha et al. 2011). PM_2.5_ was also shown to mediate dysfunctional brown adipose and mitochondrial tissues (Liu et al. 2013; Rajagopalan and Brook 2012), which is one of the systemic pathologies in T2DM (Lowell and Shulman 2005).

Chuang et al. (2010) demonstrated that exposure to air pollution [PM ≤ 10 μm (PM_10_) and ozone] exposure leads to alteration in blood pressure, blood lipids, and hemoglobin A1c, a marker of blood glucose control. Kelishadi et al. (2009) found positive associations between exposure to PM_10_, NO_2_, and insulin resistance among children in Iran. Thiering et al. (2013) later found a positive association between residential proximity to traffic, particulate matter (PM_10_), NO_2_, and risk of insulin resistance [homeostatic model assessment (HOMA-IR)] among children who were part of a birth cohort in Germany. Exposure to traffic-related air pollution is also associated with impaired glucose tolerance in pregnancy (Fleisch et al. 2014). Experimental evidence also exists for the association of air pollution and type 1 diabetes (T1DM). Ozone is known to alter T-cell dependent immune response, predisposing to autoimmune diseases (Krishna et al. 1998). It may also damage the beta cells of the pancreas possibly as a result of pulmonary reactive oxidative species production and oxidative stress, leading to reduced insulin secretion (Brenner et al. 1993; Kelishadi et al. 2009). Together with sulfate, ozone may have apoptotic properties on the beta cells (Hathout et al. 2006). The use of antioxidant prophylaxis for T1DM also points to the possibility of oxidative or inflammatory mechanisms in T1DM (Albright and Goldstein 1996).

*Strengths and limitations*. Although we applied a very broad search strategy and accepted any study design, there are few published studies on the association of air pollution with T1DM or GDM. In addition, some studies did not allow distinguishing adult T1DM from T2DM. Only three of the seven synthesized studies explicitly analyzed the T2DM risk (Coogan et al. 2012; Dijkema et al. 2011; Krämer et al. 2010). However, because > 90% of adult diabetes diagnoses are T2DM, this is unlikely to substantially affect the conclusions. Overall, the available data are not sufficient to evaluate associations with these diabetes types.

Our analysis on the association with T2DM was based on results from primary studies with unclear to high risk of bias and high diversity among the included studies. We took this into account by using effect estimates modeled to participants’ residences, converting all effect estimates to a comparable unit (per 10 μg/m^3^ of exposure), stratifying analyses by sex, including only longitudinal studies, and performing other sensitivity analyses.

The high diversity among the studies was reflected in our observation of substantial heterogeneity in the meta-analysis for NO_2_ (Table 4), which synthesized longitudinal and cross-sectional data. This was not observed for PM_2.5_, for which all studies were longitudinal. However, the number of studies was too small to further analyze this heterogeneity.

*Prospects*. Future studies should report scales of exposure assessment (pollutant quantification and traffic exposure proxies) that allow direct comparisons with existing evidence. It would be important to apply comparable models in assigning exposure to participants. Ideally, traffic distance measures should be replaced by objective particle concentration measures and models of near-road traffic-related pollutants such as ultrafine particles of elemental carbon. Also, it would be important to consider various time lags for exposure.

The studies on T1DM found associations with ozone and sulfates. These pollutants can be included in the future models for T2DM, because pollutants usually occur together in different proportions. Carbon monoxide, lead, oxidative metals, volatile organic compounds, and polycyclic aromatic hydrocarbons are other traffic-related pollutants that may be more deleterious to health but have been given less consideration.

Adjusting for noise exposure is also essential because air pollution and noise can be correlated (Foraster 2013; Kim et al. 2012; Ross et al. 2011; Tétreault et al. 2013) and share health effects. Sørensen et al. (2013) recently reported a positive association between road-traffic noise and incident diabetes, and another large meta-analysis of 10 epidemiologic studies by Cappuccio et al. (2010) found that both quality and quantity of sleep, which are related to noise, were significant predictors of the risk of T2DM. Consideration of noise is thus necessary in assessing the health effects of air pollution.

Also, socioeconomic variables should be adjusted on the spatial scale, apart from individual-level adjustment. Consideration for this spatial confounding is necessary when individual differences in health outcome are associated with neighborhood characteristics such as neighborhood socioeconomic status (Sheppard et al. 2012). It is crucial that studies on diabetes risk consider established diabetes risk factors including obesity, physical activity, and nutrition. Active and passive smoking should be considered when assessing the effect of air pollution. Lack of information on these creates a high risk for bias.

Other forms of bias such as the healthy survivor effect should be taken into account, especially in longitudinal studies. Raaschou-Nielsen et al. (2013b) demonstrated associations between diabetes mortality and NO_x_ exposure; thus, diabetes patients exposed to air pollution could die and no longer participate, resulting in incorrect estimates of association if mortality was not taken into consideration.

No included study on this topic was done in developing countries. For generalizability of evidence, research should be extended to developing countries where air pollution (including indoor) is high. This could also help in understanding effects of different air pollution compositions. Indoor air pollution is also associated with diabetes as well as cardiovascular diseases (Lee et al. 2012) and is highly prevalent in developing nations (Lim et al. 2012).

Considering the ambiguity in dose–response relationship in air pollution studies (Smith and Peel 2010), future studies should assess air pollution diabetes association in a dose–response manner. This will help in identifying the point in the dose spectrum where control will yield the most benefits for health policy (Smith and Peel 2010).

Overall, the existing evidence indicates a positive association of air pollution and T2DM risk, although there is high risk of bias. High-quality longitudinal studies are needed (taking into consideration sources and composition of air pollution as well as biomarkers) to improve our understanding of this association.

## Supplemental Material

(802 KB) PDF
