# Supplementary material for: Association between Ambient Air Pollution and Diabetes Mellitus in Europe and North America: Systematic Review and Meta-Analysis
Source: Environ Health Perspect. 2015 Jan 27;123(5):381–9. doi: 10.1289/ehp.1307823 (PMC4421762; doi:10.1289/ehp.1307823)
Supplement: (802 KB) PDF [file ehp.1307823.s001.508.pdf]

Environmental Health Perspectives doi:10.1289/ehp.1307823

Supplemental Material

Association between Ambient Air Pollution and Diabetes Mellitus in Europe and North America: Systematic Review and Meta-Analysis

Ikenna C. Eze, Lars G. Hemkens, Heiner C. Bucher, Barbara Hoffmann, Christian Schindler, Nino Künzli, Tamara Schikowski, and Nicole M. Probst-Hensch

Table S1. Association between air pollution and diabetes mellitus.

Study, exposure	Exposure contrast	Unadjusted effect estimate (95% CI)	Adjusted effect estimate (95% CI)	Confounder adjustment	Effect modification
**Krämer et al. 2010[a]**					
PM_{10}, monitoring stations	IQR: 10.1 $\mu g/m^3$	HR = 1.64 (1.20, 2.25)	HR = 1.16 (0.81, 1.65)	Baseline age, education, smoking, work place exposure to dust, fumes and extreme temperatures, BMI (average of baseline and follow-up).	Stronger association in women with high C3c levels: HR = 1.21 (0.70, 1.64)
NO_2, monitoring stations	IQR: 24.9 $\mu g/m^3$	HR = 1.53 (1.20, 1.95)	HR = 1.34 (1.02, 1.76)	Same as above	Stronger association in women with high C3c levels: HR = 1.29 (0.93, 1.79)
PM, traffic emission inventory	0.87 tons/year/km^2	HR = 1.23 (1.12, 1.35)	HR = 1.15 (1.04, 1.27)	Same as above	Stronger association in women with high C3c levels: HR = 1.24 (1.08, 1.41)
NO_2, traffic emission inventory	19 tons/year/km^2	HR = 1.22 (1.11, 1.34)	HR = 1.15 (1.04, 1.27)	Same as above	Stronger association in women with high C3c levels: HR = 1.24 (1.08, 1.41)
Soot, LUR	0.39 x 10^{-5}m	HR = 1.28 (1.12, 1.47)	HR = 1.27 (1.09, 1.48)	Same as above	Stronger association in women with high C3c levels: HR = 1.22 (1.02, 1.47)
NO_2, LUR	15 $\mu g/m^3$	HR = 1.47 (1.22, 1.77)	HR 1.42 (1.16, 1.73)	Same as above	Stronger association in women with high C3c levels: HR = 1.31 (1.01, 1.70)
Distance from a busy road	<100m	HR (low education) = 2.32 (1.29, 4.17)	HR (low education) = 2.54 (1.31, 4.91)	Same as above	Stronger association in women with high C3c levels<br>HR = 3.51 (1.50, 8.23)
Distance from a busy road	<100m	HR (high education) = 0.86 (0.55, 1.36)	HR (high education) = 0.92 (0.58, 1.47)	Same as above	Same as above
**Coogan et al. 2012[a]**					
$PM_{2.5}$	IQR: 10 $\mu g/m^3$	NA	IRR = 1.63 (0.78, 3.44)	Time-varying age, BMI, years of education, income, number of people in a household, smoking, alcohol intake, physical activity, neighbourhood socio-economic status score, family history of diabetes.	NA
NO_x	IQR: 12.4 ppb	NA	IRR = 1.25 (1.07, 1.46)	Same as above	NA

Study, exposure	Exposure contrast	Unadjusted effect estimate (95% CI)	Adjusted effect estimate (95% CI)	Confounder adjustment	Effect modification
**Andersen et al. 2012**[a]					
NO_2 (35-year mean)	IQR: 4.9 μg/m^3	HR = 1.11 (1.07, 1.15)[b]	HR = 1.04 (1.00, 1.08)	Baseline age, sex, BMI, waist-to-hip ratio, smoking status, duration and intensity, ETS, educational level, physical activity and intensity, alcohol, fruit and fat consumption and calendar year.	Stronger effects among women HR = 1.07(1.01, 1.13), subjects with high waist-to-hip ratio: HR = 1.09(1.01, 1.18), non-smokers: HR = 1.12 (1.05, 1.20), subjects with <8 years of education: HR= 1.06(1.01, 1.12), subjects with COPD: HR= 1.05(1.01, 1.09) and those with asthma: HR=1.05 (1.01, 1.09)
NO_2 (15-year mean)	IQR: 5.6 μg/m^3	HR = 1.10 (1.06, 1.13)[b]	HR = 1.04 (1.01, 1.07)	Same as above	NA
NO_2 (1-year mean at baseline)	IQR: 5.6 μg/m^3	HR = 1.08 (1.05, 1.11)[b]	HR = 1.02 (0.98, 1.05)	Same as above	NA
NO_2 (1-year mean at follow-up)	IQR: 5.7 μg/m^3	HR = 1.10 (1.06, 1.13)[b]	HR = 1.04 (1.01, 1.07)	Same as above	NA
NO_x (35-year mean)	IQR: 11.4 μg/m^3	HR = 1.05 (1.03, 1.07)[b]	HR = 1.02 (1.00, 1.04)	Same as above	NA
NO_x (15-year mean)	IQR: 12.0 μg/m^3	HR = 1.05 (1.03, 1.07)[b]	HR = 1.02 (1.00, 1.04)	Same as above	NA
NO_x (1-year mean at baseline)	IQR: 10.9 μg/m^3	HR = 1.02 (1.01, 1.02)[b]	HR = 1.00 (1.00, 1.01)	Same as above	NA
NO_x (1-year mean at follow-up)	IQR: 12.0 μg/m^3	HR = 1.05 (1.03, 1.06)[b]	HR = 1.02 (1.00, 1.04)	Same as above	NA
Traffic proximity	Major road within 50m	HR = 1.20 (1.06, 1.36)[b]	HR = 1.07 (0.95, 1.21)	Same as above	NA
Traffic load	1,300 vehicles/km/day	HR = 1.05 (1.03, 1.08)[b]	HR = 1.02 (1.00, 1.04)	Same as above	NA
**Puett et al. 2011**[a]					
$PM_{2.5}$	IQR: 4.0 μg/m^3	HR (men) = 1.05 (0.91, 1.22)[c]	HR (men) = 1.07 (0.92, 1.24)	Age, season, calendar year, state of residence, time-varying smoking status, pack years, alcohol intake, diet and hypertension, baseline BMI and physical activity.	NA
$PM_{2.5}$	IQR: 4.0 μg/m^3	HR (women) = 1.04 (0.97, 1.12)[c]	HR (women) = 1.02 (0.94, 1.09)	Same as above	NA
$PM_{2.5}$	IQR: 4.0 μg/m^3	HR (pooled) = 1.05 (0.98, 1.12)[c]	HR (pooled) = 1.03 (0.96, 1.10)	Same as above	NA
$PM_{10-2.5}$	IQR: 4.2 μg/m^3	HR (men) = 1.05 (0.94, 1.17)[c]	HR (men) = 1.04 (0.93, 1.16)	Same as above	NA
$PM_{10-2.5}$	IQR: 4.0 μg/m^3	HR (women) = 1.07 (1.01, 1.13)[c]	HR (women) = 1.04 (0.98, 1.10)	Same as above	NA
$PM_{10-2.5}$	IQR: 4.0 μg/m^3	HR (pooled) = 1.06 (1.01, 1.12)[c]	HR (pooled) = 1.04 (0.99, 1.09)	Same as above	NA
PM_{10}	IQR: 7.2 μg/m^3	HR (men) = 1.06 (0.93, 1.20)[c]	HR (men) = 1.06 (0.94, 1.20)	Same as above	NA
PM_{10}	IQR: 7.0 μg/m^3	HR (women) = 1.06 (1.01, 1.12)[c]	HR (women) = 1.03 (0.98, 1.09)	Same as above	NA
PM_{10}	IQR: 7.0 μg/m^3	HR (pooled) = 1.06 (1.01, 1.12)[c]	HR (pooled) = 1.04 (0.99, 1.09)	Same as above	NA
Distance to road	0-49m vs. ≥200m	HR (men): 0.99 (0.82, 1.19)[c]	HR (men): 1.02 (0.85, 1.23)	Same as above	NA
Distance to road	50-99m vs. ≥200m	HR (men): 0.76 (0.51, 1.14)[c]	HR (men): 0.74 (0.49, 1.11)	Same as above	NA
Distance to road	100-199m vs. ≥200m	HR (men): 0.86 (0.66, 1.13)[c]	HR (men): 0.88 (0.67, 1.16)	Same as above	NA
Distance to road	0-49m vs. ≥200m	HR (women): 1.20 (1.08, 1.33)[c]	HR (women): 1.14 (1.03, 1.27)	Same as above	Stronger effect in women
Distance to road	50-99m vs. ≥200m	HR (women): 1.20 (1.03, 1.40)[c]	HR (women): 1.16 (0.99, 1.35)	Same as above	Same as above
Distance to road	100-199m vs. ≥200m	HR (women): 1.02 (0.92, 1.14)[c]	HR (women): 0.97 (0.88, 1.08)	Same as above	Same as above
Distance to road	0-49m vs. ≥200m	HR (pooled): 1.11 (0.92, 1.33)[c]	HR (pooled): 1.11 (1.01, 1.23)	Same as above	NA
Distance to road	50-99m vs. ≥200m	HR (pooled): 0.99 (0.64, 1.54)[c]	HR (pooled): 0.96 (0.87, 1.06)	Same as above	NA
Distance to road	100-199m vs. ≥200m	HR (pooled): 0.99 (0.86, 1.13)[c]	HR (pooled): 1.02 (0.92, 1.14)	Same as above	NA

Study, exposure	Exposure contrast	Unadjusted effect estimate (95% CI)	Adjusted effect estimate (95% CI)	Confounder adjustment	Effect modification
**Brook et al. 2008[a]**					
NO_2	1 ppb	NA	OR (men) = 0.99 (0.95, 1.03)	Age, sex, BMI and neighbourhood income	NA
NO_2	1 ppb	NA	OR (women) = 1.04 (1.00, 1.08)	Same as above	Stronger effect in women
NO_2	1 ppb	NA	OR (pooled) = 1.015 (0.98, 1.049)	Same as above	Same as above
**Dijkema et al. 2011[a]**					
NO_2	14.2-15.2 vs. 8.8-14.2 $\mu g/m^3$	OR = 0.98 (0.78, 1.23)	OR = 1.03 (0.82, 1.31)	Age, sex, BMI and average monthly income	Stronger effect in women.
NO_2	15.2-16.5 vs. 8.8-14.2 $\mu g/m^3$	OR = 1.17 (0.94, 1.45)	OR = 1.25 (0.99, 1.56)	Same as above	Same as above
NO_2	16.5-36.0 vs. 8.8-14.2 $\mu g/m^3$	OR = 0.80 (0.63, 1.01)	OR = 0.80 (0.63, 1.02)	Same as above	Same as above
Distance to nearest main road	140-220m vs. 220-1610m	OR = 1.10 (0.87, 1.39)	OR = 1.12 (0.88, 1.42)	Same as above	Same as above
Distance to nearest main road	74-140m vs. 220-1610m	OR = 1.22 (0.97, 1.53)	OR = 1.17 (0.93, 1.48)	Same as above	Same as above
Distance to nearest main road	2-74m vs. 220-1610m	OR = 0.94 (0.74-1.19)	OR = 0.88 (0.70-1.13)	Same as above	Same as above
Traffic flow at the nearest main road	5871-7306 vs. 5001-5871 vehicles/day	OR = 1.09 (0.87, 1.39)	OR = 1.02 (0.81, 1.29)	Same as above	Same as above
Traffic flow at the nearest main road	7306-9670 vs. 5001-5871 vehicles/day	OR = 0.98 (0.78, 1.23)	OR = 1.03 (0.81, 1.30)	Same as above	Same as above
Traffic flow at the nearest main road	9670-35567 vs. 5001-5871 vehicles/day	OR = 0.91 (0.72, 1.16)	OR = 0.96 (0.75, 1.22)	Same as above	Same as above
Traffic in 250m buffer	516-680 x 10^3 vs. 63-516 x 10^3 vehicles/day	OR = 1.28 (1.01, 1.61)	OR = 1.25 (0.99, 1.59)	Same as above	Same as above
Traffic in 250m buffer	680-882 x 10^3 vs. 63-516 x 10^3 vehicles/day	OR = 1.15 (0.91, 1.46)	OR = 1.13 (0.89, 1.44)	Same as above	Same as above
Traffic in 250m buffer	882-2007 x 10^3 vs. 63-516 x 10^3 vehicles/day	OR = 1.13 (0.89, 1.44)	OR = 1.09 (0.85, 1.38)	Same as above	Same as above
**Chen et al. 2013[a]**					
$PM_{2.5}$	10 $\mu g/m^3$	HR = 1.08 (0.99, 1.17)[d]	HR = 1.11 (1.02, 1.21)	Baseline age, sex survey year, region, marital status, education, household income, BMI, physical activity, smoking, alcohol consumption, diet, race, hypertension, urban residency, neighbourhood-level unemployment rate, education, COPD, asthma, congestive heart failure and acute myocardial infarction	Stronger effects among subjects with COPD: HR= 1.33 (1.03, 1.71), women: HR= 1.17 (1.03, 1.32), subjects aged<50 years: HR= 1.19 (1.00, 1.40) or >65 years: HR= 1.18 (1.01, 1.38) and subjects with low level of education: HR= 1.13 (1.00, 1.28).
**van den Hooven et al. 2009**					
Distance-weighted traffic density	158-546 vs. <158 vehicles/ day*km	OR = 0.66 (0.30, 1.48)	OR = 0.69 (0.30, 1.57)	Maternal age, education, ethnicity, BMI, parity, smoking, alcohol consumption, month and year of birth.	NA
Distance-weighted traffic density	546-1,235 vs. <158 vehicles/ day*km	OR = 1.00 (0.49, 2.05)	OR = 1.07 (0.51, 2.23)	Same as above	NA
Distance-weighted traffic density	>1,235 vs. <158 vehicles/ day*km	OR = 0.67 (0.30, 1.49)	OR = 0.79 (0.35, 1.81)	Same as above	NA
Distance to major road	150-200m vs. >200m	OR = 1.17 (0.53, 2.60)	OR = 1.07 (0.47, 2.44)	Same as above	NA
Distance to major road	100-150m vs. >200m	OR = 0.76 (0.32, 1.82)	OR = 0.77 (0.32, 1.88)	Same as above	NA

Study, exposure	Exposure contrast	Unadjusted effect estimate (95% CI)	Adjusted effect estimate (95% CI)	Confounder adjustment	Effect modification
Distance to major road	50-100m vs. >200m	OR = 1.07 (0.50, 2.31)	OR = 1.13 (0.51, 2.50)	Same as above	NA
**Malmqvist et al. 2013**					
NO_x	9.0-14.1 vs. 2.5-8.9 μg/m^3	OR = 1.28 (1.07, 1.54)	OR = 1.19 (0.99, 1.44)	Maternal age, parity, prepregnancy BMI, calendar year, ethnicity, T1DM	NA
NO_x	14.2-22.6 vs. 2.5-8.9 μg/m^3	OR = 1.84 (1.56, 2.18)	OR = 1.52 (1.28, 1.82)	Same as above	NA
NO_x	>22.7 vs. 2.5-8.9 μg/m^3	OR = 1.98 (1.68, 2.35)	OR = 1.69 (1.41, 2.03)	Same as above	NA
Traffic density within 200m	<2 cars/min vs. No road	OR = 0.89 (0.75, 1.06)	OR = 0.93 (0.78, 1.12)	Same as above	NA
Traffic density within 200m	2-5 cars/min vs. No road	OR = 1.04 (0.88, 1.23)	OR = 0.96 (0.81, 1.14)	Same as above	NA
Traffic density within 200m	5-10 cars/min vs. No road	OR = 1.53 (1.27, 1.84)	OR = 1.18 (0.97, 1.43)	Same as above	NA
Traffic density within 200m	>10 cars/min vs. No road	OR = 1.50 (1.24, 1.82)	OR = 1.23 (1.01, 1.51)	Same as above	NA
**Hathout et al 2006**					
O_3	10 ppb	OR = 2.92 (1.86, 4.58)	OR = 1.73 (1.08, 2.77)	Age at diagnosis/entry, ETS, attendance of day care, breast feeding, maternal diabetes, family history of diabetes and autoimmunity, maternal drug use, parental education.	NA
SO_4	10 μg/m^3	OR = 1.65 (1.20, 2.28)	NA	NA	NA
SO_2	1 ppb	OR = 1.42 (0.91, 2.21)	NA	NA	NA
NO_2	10 ppb	OR = 1.03 (0.71, 1.50)	NA	NA	NA
PM_{10}	10 μg/m^3	OR = 1.08 (0.87, 1.34)	NA	NA	NA
**Hathout et al 2002**					
O_3	IQR: 10.93 ppb	OR = 4.22 (1.96, 9.10)	OR = 4.22 (1.96, 9.10)	Age	NA
SO_4	IQR: 1.025 μg/m^3	OR = 0.56 (0.37, 0.87)	OR = 0.55 (0.35, 0.85)	Same as above	NA
SO_2	IQR: 1.235 ppb	OR = 0.54 (0.33, 0.89)	OR = 0.52 (0.31, 0.88)	Same as above	NA
NO_2	IQR: 11.175 ppb	OR = 0.57 (0.31, 1.02)	OR = 0.56 (0.30, 1.03)	Same as above	NA
PM_{10}	IQR: 22.65 μg/m^3	OR = 2.37 (1.11, 5.03)	OR = 2.37 (1.11, 5.03)	Same as above	NA
**Fleisch et al. 2014**					
Central-site $PM_{2.5}$	IQR: 1.7 μg/m^3	NA	OR = 0.81 (0.62, 1.08)	Age, prepregnancy BMI, pregnancy weight gain, education, race/ethnicity, family history of diabetes, prior GDM and season of last menstrual period.	NA
Central-site $PM_{2.5}$	10.0-10.7 vs. 8.3-10.0 μg/m^3	NA	OR = 0.91 (0.50, 1.65)	Same as above	NA
Central-site $PM_{2.5}$	10.7-11.7 vs. 8.3-10.0 μg/m^3	NA	OR = 0.52 (0.27, 1.00)	Same as above	NA
Central-site $PM_{2.5}$	11.7-17.2 vs. 8.3-10.0 μg/m^3	NA	OR = 0.69 (0.38, 1.27)	Same as above	NA
Spatiotemporal $PM_{2.5}$	IQR: 2.0 μg/m^3	NA	OR = 0.94 (0.67, 1.34)	Same as above	NA
Spatiotemporal $PM_{2.5}$	10.8-11.8 vs. 8.5-10.8 μg/m^3	NA	OR = 0.62 (0.30, 1.28)	Same as above	NA
Spatiotemporal $PM_{2.5}$	11.8-12.8 vs. 8.5-10.8 μg/m^3	NA	OR = 0.93 (0.48, 1.78)	Same as above	NA
Spatiotemporal $PM_{2.5}$	12.8-15.9 vs. 8.5-10.8 μg/m^3	NA	OR = 0.71 (0.35, 1.42)	Same as above	NA
Central-site black carbon	IQR: 0.16 μg/m^3	NA	OR = 0.69 (0.42, 1.13)	Same as above	NA
Central-site black carbon	0.78-0.87 vs. 0.60-0.78 μg/m^3	NA	OR = 0.75 (0.39, 1.45)	Same as above	NA

Study, exposure	Exposure contrast	Unadjusted effect estimate (95% CI)	Adjusted effect estimate (95% CI)	Confounder adjustment	Effect modification
Central-site black carbon	0.87-0.94 vs. 0.60-0.78 $\mu g/m^3$	NA	OR = 0.59 (0.25, 1.35)	Same as above	NA
Central-site black carbon	0.94-1.10 vs. 0.60-0.78 $\mu g/m^3$	NA	OR = 0.60 (0.23, 1.53)	Same as above	NA
Spatiotemporal black carbon	IQR: 0.34 $\mu g/m^3$	NA	OR = 1.02 (0.73, 1.41)	Same as above	NA
Spatiotemporal black carbon	0.55-0.70 vs. 0.14-0.55 $\mu g/m^3$	NA	OR = 1.01 (0.54, 1.87)	Same as above	NA
Spatiotemporal black carbon	0.70-0.89 vs. 0.14-0.55 $\mu g/m^3$	NA	OR = 1.12 (0.59, 2.09)	Same as above	NA
Spatiotemporal black carbon	0.89-1.69 vs. 0.14-0.55 $\mu g/m^3$	NA	OR = 0.90 (0.45, 1.79)	Same as above	NA
Neighbourhood traffic density within 100m	IQR: 1,533 vehicles/day*km	NA	OR = 1.02 (0.87, 1.18)	Same as above	NA
Neighbourhood traffic density within 100m	4,062-9,680 vs. 0-4,061 vehicles/day*km	NA	OR = 1.18 (0.66, 2.11)	Same as above	NA
Neighbourhood traffic density within 100m	9,680-19,371vs. 0-4,061 vehicles/day*km	NA	OR = 0.94 (0.51, 1.72)	Same as above	NA
Neighbourhood traffic density within 100m	19,383-30,860 vs. 0-4,061 vehicles/day*km	NA	OR = 0.74 (0.39, 1.42)	Same as above	NA
Home roadway proximity	≤200m vs. >200m	NA	OR = 0.99 (0.52, 1.88)	Same as above	NA
**Pearson et al. 2010**					
$PM_{2.5}$ (36km model, 2004)	10 $\mu g/m^3$	OR = 6.69 (5.53, 7.77)	OR = 3.16 (2.77, 3.74)	County-level median age, per capita income, percentage of men, per capita income, percentage of the population aged >25 years with a high school or general equivalency degree, percentage of ethnicities, prevalence of obesity, physical activity, population density and latitude (from census 2000)	NA
$PM_{2.5}$ (36km model, 2004)	10 $\mu g/m^3$	OR = 6.69 (5.53, 7.77)	OR = 2.18 (1.48, 3.49)	Same as above (from ACS 1-year)	NA
$PM_{2.5}$ (36km model, 2005)	10 $\mu g/m^3$	OR = 6.69 (5.42, 7.92)	OR = 2.51 (2.12, 3.10)	Same as above (from census 2000)	NA
$PM_{2.5}$ (36km model, 2005)	10 $\mu g/m^3$	OR = 6.69 (5.42, 7.92)	OR = 2.25 (1.62, 2.91)	Same as above (from ACS 1-year)	NA

PM: particulate matter; PM_{10}: particulate matter <10µm in diameter; $PM_{10-2.5}$: particulate matter between 2.5 and 10µm in diameter; $PM_{2.5}$: particulate matter <2.5µm in diameter; NO_2: nitrogen dioxide; NO_x: nitrogen oxides; O_3: ozone; SO_2: sulphur dioxide; SO_4: sulphate; T1DM: type 1 diabetes mellitus; GDM: gestational diabetes mellitus; LUR: land-use regression; IQR: interquartile range; C3c: complement protein 3c; ETS: environmental tobacco smoking; BMI: body mass index; COPD: chronic obstructive pulmonary disease; NA: not available; ACS: American Community Survey.

[a]Included in meta-analysis. [b]Adjusted for only age. [c]Adjusted for age, season and year. [d]Adjusted for age, sex, year and region.

Table S2. Risk of bias assessment for included studies.

Source	Adjustment for basic DM risk factors[a] at baseline	Exposure assessment before DM diagnosis	Exposure modelled at participants' residence	Attempts to identify undiagnosed DM	Consideration of healthy survivor bias	Adjustment for noise as an environmental risk factor	Consideration of time-dependent confounding
Krämer et al. 2010[b]	Yes[c]	Yes	Yes	No	No	No	No
Andersen et al. 2012[b]	Yes[c]	Yes	Yes	No	No	No	Yes
Puett et al. 2011[b]	Yes[c]	Yes	Yes	No	No	No	Yes
Coogan et al. 2012[b]	Yes[d]	No	Yes	No	No	No	Yes
Chen et al. 2013[b]	Yes[b]	Yes	Yes	No	No	No	No
Brook et al. 2008[b]	Yes[e]	No	Yes	No	NA	No	NA
Dijkema et al. 2011[b]	Yes[f]	No	Yes	Yes	NA	No	NA
Pearson et al. 2010	Yes[g]	NA	NA	NA	NA	No	NA
Malmqvist et al. 2013	Yes[c]	No	Yes	No	NA	No	NA
Van den Hooven et al. 2009	Yes[c]	Yes	Yes	No	NA	No	NA
Hathout et al. 2002	Yes[h]	Yes	Yes	No	NA	No	NA
Hathout et al. 2006	Yes	Yes	Yes	Yes	NA	No	NA
Fleisch et al. 2014	Yes[d]	No	Yes	Yes	NA	No	NA

[a]Include age, BMI, socio-economic status, smoking, family history and physical activity. [b]Included in meta-analysis. [c]Except family history. [d]Except physical activity. [e]Except family history, physical activity; [f]except family history, physical activity, smoking. [g]On ecologic scale. [h]Only age. NA: not applicable.

References

Andersen ZJ, Raaschou-Nielsen O, Ketzel M, Jensen SS, Hvidberg M, Loft S, et al. 2012. Diabetes incidence and long-term exposure to air pollution: A cohort study. Diabetes Care 35:92-98.

Brook RD, Jerrett M, Brook JR, Bard RL, Finkelstein MM. 2008. The relationship between diabetes mellitus and traffic-related air pollution. J Occup Environ Med 50:32-38.

Chen H, Burnett RT, Kwong JC, Villeneuve PJ, Goldberg MS, Brook RD, et al. 2013. Risk of incident diabetes in relation to long-term exposure to fine particulate matter in Ontario, Canada. Environ Health Perspect 121:804-810.

Coogan PF, White LF, Jerrett M, Brook RD, Su JG, Seto E, et al. 2012. Air pollution and incidence of hypertension and diabetes mellitus in black women living in Los Angeles. Circulation 125:767-772.

Dijkema MB, Mallant SF, Gehring U, van den Hurk K, Alssema M, van Strien RT, et al. 2011. Long-term exposure to traffic-related air pollution and type 2 diabetes prevalence in a cross-sectional screening-study in the Netherlands. Environ Health 10:76.

Fleisch AF, Gold DR, Rifas-Shiman SL, Koutrakis P, Schwartz JD, Kloog I, et al. 2014. Air pollution exposure and abnormal glucose tolerance during pregnancy: The project Viva cohort. Environ Health Perspect 122:378-383.

Hathout EH, Beeson WL, Nahab F, Rabadi A, Thomas W, Mace JW. 2002. Role of exposure to air pollutants in the development of type 1 diabetes before and after 5 yr of age. Pediatr Diabetes 3:184-188.

Hathout EH, Beeson WL, Ischander M, Rao R, Mace JW. 2006. Air pollution and type 1 diabetes in children. Pediatr Diabetes 7:81-87.

Krämer U, Herder C, Sugiri D, Strassburger K, Schikowski T, Ranft U, et al. 2010. Traffic-related air pollution and incident type 2 diabetes: Results from the SALIA cohort study. Environ Health Perspect 118:1273-1279.

Malmqvist E, Jakobsson K, Tinnerberg H, Rignell-Hydbom A, Rylander L. 2013. Gestational diabetes and preeclampsia in association with air pollution at levels below current air quality guidelines. Environ Health Perspect 121:488-493.

Pearson JF, Bachireddy C, Shyamprasad S, Goldfine AB, Brownstein JS. 2010. Association between fine particulate matter and diabetes prevalence in the U.S. Diabetes Care 33:2196-2201.

Puett RC, Hart JE, Schwartz J, Hu FB, Liese AD, Laden F. 2011. Are particulate matter exposures associated with risk of type 2 diabetes? Environ Health Perspect 119:384-389.

van den Hooven EH, Jaddoe VW, de Kluizenaar Y, Hofman A, Mackenbach JP, Steegers EA, et al. 2009. Residential traffic exposure and pregnancy-related outcomes: A prospective birth cohort study. Environ Health 8:59.
